# Kidney Dysfunction in Adult Offspring Exposed In Utero to Type 1 Diabetes Is Associated with Alterations in Genome-Wide DNA Methylation

**DOI:** 10.1371/journal.pone.0134654

**Published:** 2015-08-10

**Authors:** Jean-François Gautier, Raphaël Porcher, Charbel Abi Khalil, Naima Bellili-Munoz, Lila Sabrina Fetita, Florence Travert, Simeon-Pierre Choukem, Jean-Pierre Riveline, Samy Hadjadj, Etienne Larger, Philippe Boudou, Bertrand Blondeau, Ronan Roussel, Pascal Ferré, Eric Ravussin, François Rouzet, Michel Marre

**Affiliations:** 1 Department of Diabetes and Endocrinology, Assistance Publique—Hôpitaux de Paris, DHU FIRE, Lariboisière Hospital, University Paris-Diderot Paris-7, Paris, France; 2 Clinical Investigation Center, INSERM-CIC9504, Saint-Louis University Hospital, Assistance Publique—Hôpitaux de Paris, University Paris-Diderot Paris-7, Paris, France; 3 INSERM UMRS 1138, Cordeliers Research Center, University Pierre et Marie Curie Paris-6, Paris, France; 4 Department of Biostatistics and Medical Computing, Saint-Louis University Hospital, Assistance Publique—Hôpitaux de Paris, University Paris-Diderot Paris-7, Paris, France; 5 Department of Diabetes, Groupe Hospitalier Bichat—Claude Bernard, Assistance Publique—Hôpitaux de Paris, DHU FIRE, University Paris-Diderot Paris-7, Paris, France; 6 Clinical Investigation Center, Groupe Hospitalier Bichat—Claude Bernard, Assistance Publique–Hôpitaux de Paris, University Paris-Diderot Paris-7, Paris, France; 7 Department of Diabetes and Endocrinology, Centre Hospitalier Sud Francilien, Corbeil-Essonnes, France; 8 Department of Endocrinology and Diabetes, Centre Hospitalier Universitaire, Poitiers, France; 9 Department of Diabetes, Hôtel-Dieu Hospital, Assistance Publique–Hôpitaux de Paris, Paris, France; 10 Unit of Transfer in Molecular Oncology and Hormonology, Saint-Louis University Hospital, Assistance Publique—Hôpitaux de Paris, Paris, France; 11 INSERM U695, University Paris-Diderot Paris-7, Paris, France; 12 Penington Biomedical Research Center, Baton Rouge, LA, United States of America; 13 Department of Nuclear Medicine, Groupe Hospitalier Bichat—Claude Bernard, Assistance Publique—Hôpitaux de Paris, University Paris-Diderot Paris-7, Paris, France; University of Bristol, UNITED KINGDOM

## Abstract

**Background:**

Fetal exposure to hyperglycemia impacts negatively kidney development and function.

**Objective:**

Our objective was to determine whether fetal exposure to moderate hyperglycemia is associated with epigenetic alterations in DNA methylation in peripheral blood cells and whether those alterations are related to impaired kidney function in adult offspring.

**Design:**

Twenty nine adult, non-diabetic offspring of mothers with type 1 diabetes (T1D) (case group) were matched with 28 offspring of T1D fathers (control group) for the study of their leukocyte genome-wide DNA methylation profile (27,578 CpG sites, Human Methylation 27 BeadChip, Illumina Infinium). In a subset of 19 cases and 18 controls, we assessed renal vascular development by measuring Glomerular Filtration Rate (GFR) and Effective Renal Plasma Flow (ERPF) at baseline and during vasodilatation produced by amino acid infusion.

**Results:**

Globally, DNA was under-methylated in cases vs. controls. Among the 87 CpG sites differently methylated, 74 sites were less methylated and 13 sites more methylated in cases vs. controls. None of these CpG sites were located on a gene known to be directly involved in kidney development and/or function. However, the gene encoding DNA methyltransferase 1 (DNMT1)—a key enzyme involved in gene expression during early development–was under-methylated in cases. The average methylation of the 74 under-methylated sites differently correlated with GFR in cases and controls.

**Conclusion:**

Alterations in methylation profile imprinted by the hyperglycemic milieu of T1D mothers during fetal development may impact kidney function in adult offspring. The involved pathways seem to be a nonspecific imprinting process rather than specific to kidney development or function.

## Introduction

Fetal programming defines a phenomenon by which an alteration of intrauterine environment predisposes to the development of disorders later in life. Evidence came from epidemiological data showing that tobacco exposure, caloric restriction, and severe hyperglycemia during pregnancy as well as severe pregnancy-related hypertension are all determinants of low birth weight [1], which is associated with higher prevalence of metabolic and cardiovascular disorders at adult age (review in [2]). Hyperglycemia during pregnancy independently of birth weight is also associated with higher risks of cardio-metabolic disease later in life [3–7].

A reduced number of nephrons has been associated with primary hypertension and renal and cardiovascular risks in humans [8, 9]. The number of nephrons is determined at birth in humans [10] and is correlated to birth weight [11], supporting a role of intrauterine environment on kidney function at adult age.

We previously investigated adult individuals born from type 1 diabetic mothers as a model of fetal exposure to maternal hyperglycemia, taking those born from type 1 diabetic fathers as controls [12, 13]. Hence the genetic background in relation to type 1 diabetes is equivalent in exposed subjects and controls. Data obtained in offspring of type 2 diabetic mothers (4) can be obscured by the backgrounds of essential hypertension, or other components of the insulin resistance syndrome so frequently associated with type 2 diabetes. Then, we were able to investigate the impact of hyperglycemia per se on kidney function. We found that fetal exposure to maternal type 1 diabetes is associated at adult age with a reduced functional reserve by measuring glomerular filtration rate and effective renal plasma flow [13], a surrogate of functional nephron numbers [8]. This finding was consistent with studies in a rat model in which moderate hyperglycemia during pregnancy was associated with a decreased number of nephrons in offspring [14], which favored the development of hypertension at adulthood [15].

The molecular mechanisms involved in the development of impaired renal function in offspring of diabetic mothers are yet to be unravelled. Alterations of the expressions of IGFs and their receptors in fetal kidney were reported in rats exposed in utero to maternal hyperglycemia [16]. Because of its impact on kidney development, abnormal angiogenesis may be a plausible mechanism [17, 18]. In a classical model of angiogenesis, we observed that high glucose levels induce a defect in angiogenesis by increasing apoptosis and reducing proliferation of endothelial cells without affecting the expression of several growth factors involved in angiogenesis [19].

If adverse fetal environment irreversibly programs adult disease, then DNA epigenetic modifications may be involved. DNA methylations and histone modifications are two key epigenetic changes in chromatin structure that directly influence gene transcription, expression, and cellular functions and consequently, tissue development [20]. DNA methylation, the most studied transcriptional epigenetic modification, is characterized by the covalent addition of a methyl group on a cytosine base by DNA methyl transferases in CpG dinucleotides (CpG sites). When CpG methylation occurs in promoter regions of genes, transcription is usually decreased. It may be thus involved in gene silencing, genomic imprinting, and chromosomal stability [21] and consequently be a potential mechanism linking hyperglycemia exposure during fetal development and renal dysfunction in adulthood [20]. DNA methylation may vary depending on cell type so that DNA methylation profile may differ from one organ to another, except if DNA methylation changes occurred very early in the fetal development.

In the present study we used a microarray technique to seek potential alterations in genome-wide DNA methylation in blood cells from adult subjects who had been exposed in utero to maternal type 1 diabetes. We also investigated whether these alterations are associated with renal dysfunction.

## Materials and Methods

### Subjects

Participants were direct offspring of type 1 diabetic subjects attending specialized clinics in 6 French hospitals: Hôpital Saint-Louis, Hôpital Bichat–Claude Bernard, Hôtel-Dieu, Institut Montsouris all in Paris, Centre Hospitalier Universitaire in Poitiers, and Centre Hospitalier Sud Francilien in Corbeil, Essonne.

Cases and controls were selected to have one parent with type 1 diabetes (as defined by the American Diabetes Association) for at least two years before offspring conception. Eligibility was possible if the other parent was not diabetic at time of study. All mothers did not smoke during pregnancy. Offspring were men or women at least 18 years of age not pregnant at time of investigation for women and without diabetes as checked with an oral glucose tolerance test. They were free of immune marker of type 1 diabetes (anti-islet antibodies, antibodies against GAD, IA2, and IA2 beta, and anti-insulin antibodies). Chronic drug intake, acute infection, any chronic disease, and personal or family history of kidney disease, other than possible diabetic nephropathy in their diabetic parents were excluded. Cases were offspring of type 1 diabetic mothers, and controls offspring of type 1 diabetic fathers.

The current study is part of a research program designed to investigate the physiological consequences of fetal exposure to maternal type 1 diabetes at adult age in 62 offspring recruited between 2006 and 2009. Genomic DNA isolated from whole blood samples was available for CpG sites methylation assay in 57 offspring, 29 cases and 28 controls. Among them, 19 cases and 18 controls were studied for baseline and amino acid-stimulated Glomerular Filtration Rate (GFR), and Effective Renal Plasma Flow (ERPF), using a 51Cr EDTA plus 123I-hippurate primed constant infusion technique as previously described [13].

### Ethics statement

The study was approved by the local Ethical Committee (Comité Consultatif de Protection des Personnes dans la Recherche Biomédicale de Paris Saint-Louis; AOR 04032) and each participant gave a written informed consent to participate.

### Genome-wide DNA methylation analysis

Methylation of 27,578 CpG sites at 14,475 consensus coding sequencing sites was performed using the Illumina Human Methylation27 BeadChip system at the Integragen SA at Evry, France, as previously described [22, 23]. Briefly, 4 ml of bisulfite converted DNA was amplified overnight at 37°C. The amplified DNA product was fragmented by an endpoint enzymatic process. Fragmented DNA was precipitated, resuspended, and applied to an Infinium Human Methylation27 BeadChip and hybridized overnight. During hybridization, the amplified and fragmented DNA samples anneal to specific oligomers that are covalently linked to over 27,000 different bead types. Each bead type corresponds to the nucleotide identity and thus the methylation status at a bisulfite-converted cytosine in a specific CpG site. The bead chips were then subjected to a single-base extension reaction using the hybridized DNA as a template incorporating fluorescently labelled nucleotides of two different colors, each corresponding to the cytosine (methylated) or uracil (unmethylated) identity of the bisulfite-converted nucleotide. The fluorescently stained chip was imaged by the Illumina BeadArray Reader. Illumina’s Genome Studio program was used to analyze BeadArray data to assign site-specific DNA methylation β-values to each CpG site. The β-value defined the proportion of methylation for each subject at each CpG site which was computed by first subtracting the background signal intensity of negative controls from both the methylated and unmethylated signals and then taking the ratio of the methylated signal intensity to the sum of both methylated and unmethylated signals. Thus, the β-value is a continuous variable ranging between 0 and 1.

### Data analyses

#### Quality control

The detection p-value as provided by Illumina is obtained by comparing the signal generated by each CpG site to negative controls. CpG sites with missing β-values or detection p-value > 0.01 for more than 5 patients were eliminated from the analysis. This strategy led to discard 1509 CpG sites (5.5%), thus leaving 26,069 CpG sites among 57 subjects for the analysis.

**Fig 1:**
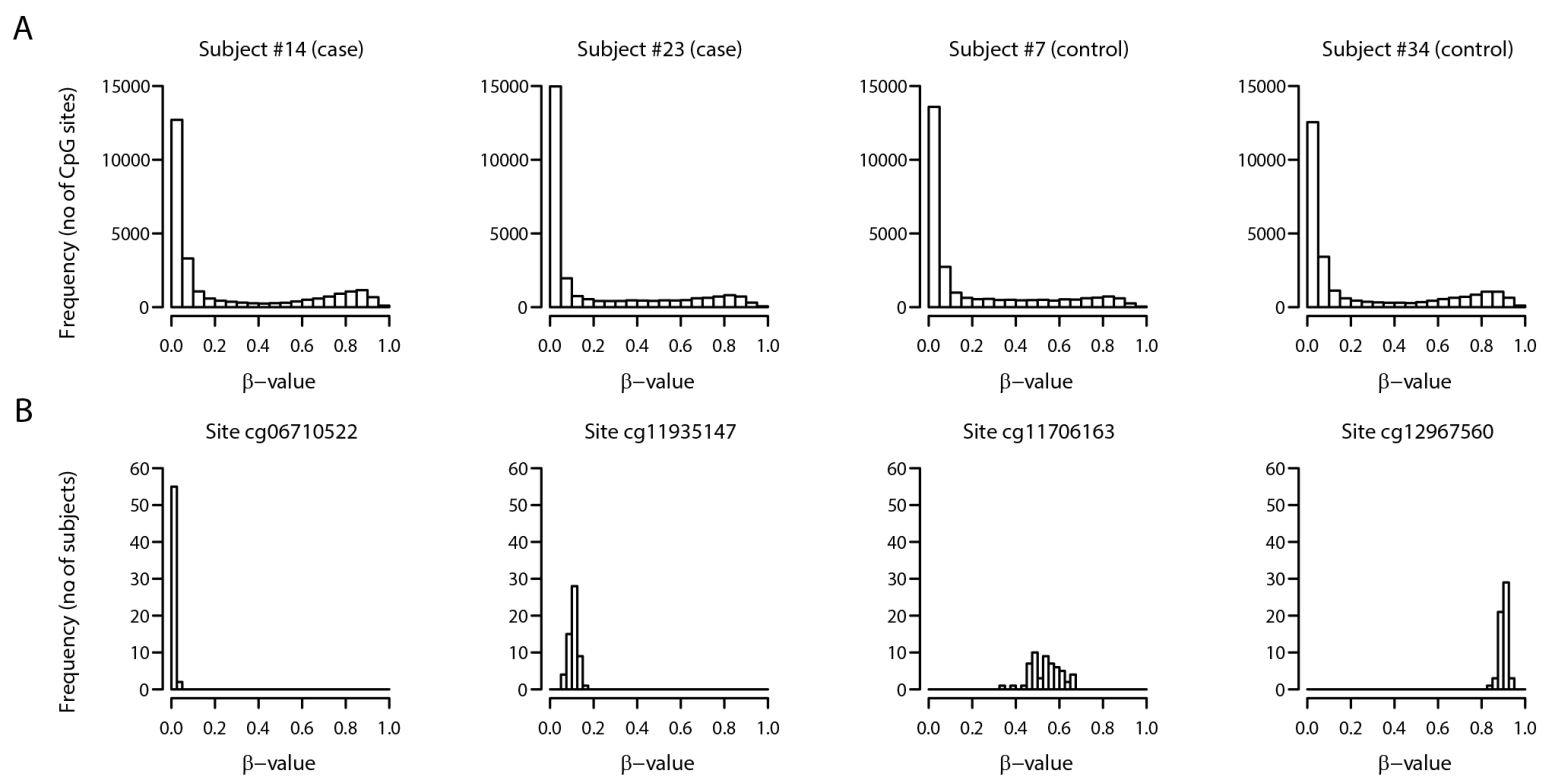
Distribution of β-values for selected subjects (cases and controls) and CpG sites. Panel A: distribution of β-values for 26,069 CpG sites for four randomly selected subjects (two among cases, and two among control subjects). Panel B: Distribution of β-values at selected CpG sites for all 57 study subjects.

Fig 1 illustrates the methylation profile of 4 randomly selected subjects (panel A), showing that individuals have a peak of low methylated CpG sites, a smaller peak of high methylated sites, and a small proportion of moderately methylated sites. As an example, Panel B depicts the distributions of methylations (β-value) at selected CpG sites for the 57 subjects.

#### Determination of differentially methylated CpG sites between cases and controls

After initial quality control as described above, a sample of 57 subjects (29 cases and 28 controls) was tested for association between fetal exposure status and gene-specific methylation. In this analysis, the logit-transformed β-value of each CpG site was compared between cases and controls using t-tests. Then, p-values were obtained from *B* = 100 permutations of the t-statistics as described in Storey & Tibshirani [24, 25]. More precisely, the exposure status of subjects were randomly permuted *B* = 100 times, thus leading to 100 samples where no association between methylation at each CpG site and exposure is expected (because exposure was mixed across subjects at random). For each CpG site *j*, the new artificial “case” and “control” groups were compared, leading to t-statistics ${\text{t}}_{\text{j}}^{0\text{b}}$ $t_{j}^{0b}$, *b* = 1,…,*B*. For a given CpG site *i*, *i* = 1,…,n_CpG_ *n*_*CpG*_ (= 26,069), the p-value *p*_*i*_ was calculated as

$$p_{i}=\sum_{b=1}^{B}\frac{\#\left\{\text{j}:\left|{\text{t}}_{\text{j}}^{0\text{b}}\right|\geq \left|t_{i}\right|,j=1,\ldots ,n_{CpG}\right\}}{n_{CpG}\times B}.$$

CpG sites were considered as differentially methylated if they achieved a p-value below the pre-specified arbitrary threshold of 0.005. Cut-off p-values commonly used in similar DNA methylation chip studies are set between 0.05 and 0.005 [23, 26]. Average methylation of up-methylated and down-methylated sites in cases was then computed and its association with renal parameters analyzed using multiple regression models with the renal parameter as dependent variable and the group (case or control), the average methylation and their interaction as independent variables.

Analyses were performed using the R statistical programming software (the R foundation for Statistical Computing, Vienna, Austria)

#### Identification of biological processes

Further analysis of the differentially methylated genes was conducted for potential biological significance using an automated method of literature interrogation, the Acumenta Literature Lab: it identifies and ranks associations existing in the literature between gene sets, such as those derived from microarray experiments, and curated sets of key terms such as pathway names, medical subject heading [27]. First, the software was questioned with known pathways or genes involved in kidney development and function such as IGF2, angiogenesis, renin angiotensin system or in renal disease risk such as APOL1 and MYH9 [28]. In a second step, we ran the software without a priori.

#### Methylation specific PCR

In order to validate DNA methylation changes detected by the genome-wide analysis from Illumina Chip, we performed methylation specific PCR (Epitect, Qiagen) for some most differentially methylated genes accordingly to the manufacturer instructions.

## Results

**Fig 2:**
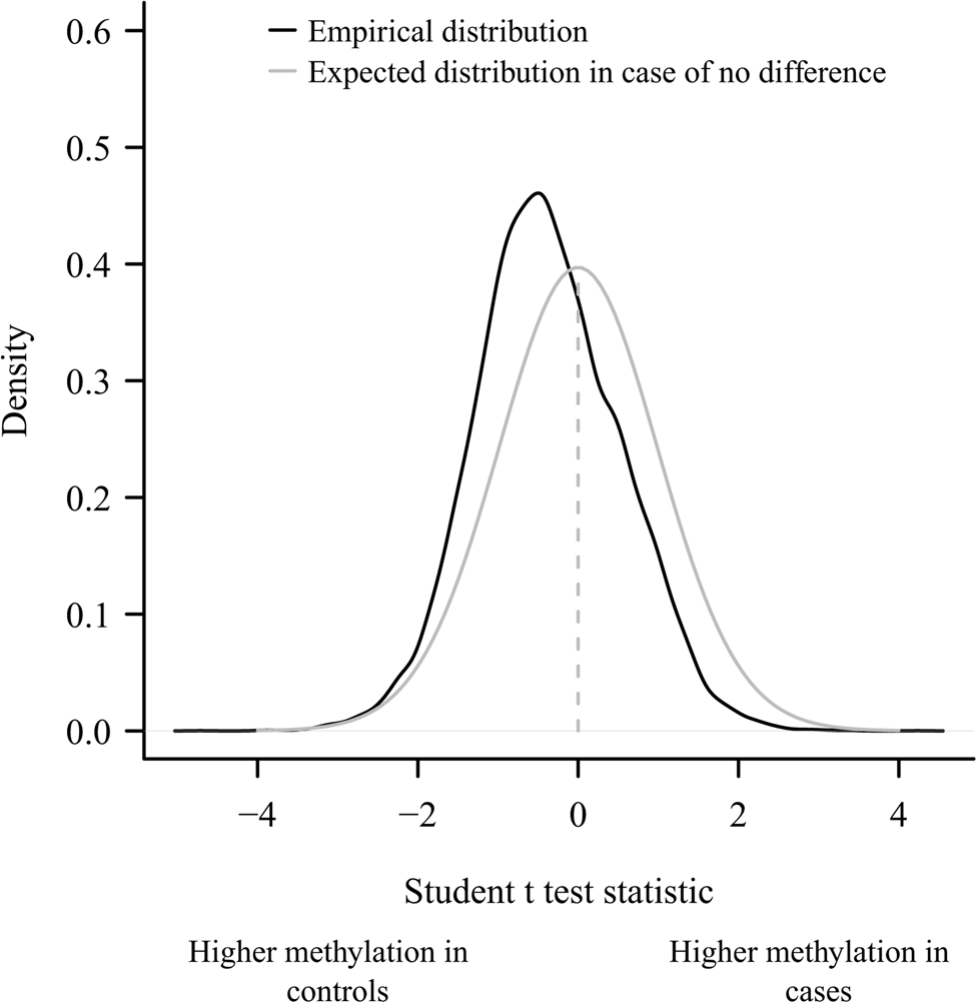
Distribution of the t-test statistic. Distribution of the t-test statistic when comparing the β-value of the 26,069 methylation sites between offspring of diabetic fathers (controls) and of diabetic mothers (cases). The mean of the test is not zero (*p<10* ^*−12*^) as expected in case of no difference between the 2 groups.

Characteristics of parents and offspring are shown in Table 1. They were similar in the 2 groups except for the prevalence of late prematurity (born between 34 and 37 weeks of pregnancy) which was higher in the offspring of diabetic mothers (cases) than in offspring of diabetic fathers (controls). There was no preterm delivery less than 34 weeks of pregnancy and only one case was born at 34 weeks. In order to seek differences in methylation profile between case and controls, we looked at the distribution of t-statistics at each CpG sites. We found that the observed distribution differed from its expectation in case of no difference in DNA methylation between case and controls, and was shifted in favor of higher methylation in controls (Fig 2).

**Table 1:** Characteristics of parents and offspring.

	Controls	Cases	p
	(n = 28)	(n = 29)	
Diabetic parent characteristics			
Sex, men/women	28/0	0/29	
Current age, y	56.7 (6.0)	54.2 (6.6)	0.14
Age at diabetes onset, y	17.8 (8.5)	15.5 (8.0)	0.31
Age at offspring birth, y	31.2 (4.8)	27.9 (3.7)	0.006
Current Body mass index, kg/m²	25.5 (3.9)	25.2 (4.1)	0.81
Nephropathy, no. (%)	5 (19)	4 (14)	>0.99
Retinopathy, no. (%)	24 (86)	20 (71)	0.33
Macroangiopathy, no. (%)	6 (21)	5 (19)	>0.99
Birth data			
Preterm delivery, no. (%)	0 (0)	10 (36)	0.003
Birthweight, g	3354 (474)	3282 (661)	0.66
Offspring clinical characteristics			
Female gender, no. (%)	14(50)	16 (55)	0.79
Age, y	25.6 (5.0)	25.9 (6.2)	0.85
Body mass index, kg/m²	22.8 (2.8)	23.2 (3.2)	0.63
Systolic blood pressure, mm Hg	122 (13)	123 (14)	0.85
Diastolic blood pressure, mm Hg	67 (10)	69 (9)	0.52
Body fat, %	24.6 (8.0)	26.3 (8.7)	0.46
Men	19.0 (6.1)	19.5 (5.9)	0.86
Women	30.1 (5.4)	31.8 (6.3)	0.47
Waist circumference, cm	80 (9)	77 (9)	0.28
Men	84 (8)	82 (11)	0.73
Women	77 (8)	74 (6)	0.43
Offspring biological characteristics			
Serum creatinine, μmol/L	75 (11)	74 (14)	0.78
Uricemia, μmol/L	297 (86)	284 (72)	0.55
Total cholesterol, mmol/L	4.5 (0.9)	4.9 (1.0)	0.093
Triglycerides, mmol/L	0.95 (0.43)	0.98 (0.39)	0.79
LDL cholesterol, mmol/L	2.6 (0.7)	3 (1.1)	0.16
HDL cholesterol, mmol/L	1.5 (0.3)	1.7 (0.6)	0.14

Mean (SD) otherwise stated.

As shown in Table 2, 87 CpG sites were differentially methylated between cases and controls. Among them, 74 were down-methylated and 13 sites were up-methylated in cases vs controls.

**Table 2:** Details on the 87 differentially methylated sites ranked by p-value. Direction (+) indicates higher methylation in cases and (-) in controls.

SYMBOL	Gene name	Index	p-value	Direction
FCN1	ficolin 1 precursor	17386	1.07E-05	-
ERMAP	erythroblast membrane-associated protein	17353	6.14E-05	+
MUC5B	mucin 5; subtype B; tracheobronchial	22377	0.00016	-
CYP4F3	cytochrome P450; family 4; subfamily F; polypeptide 3	16455	0.00020	-
TMBIM1	PP1201 protein	25876	0.00023	-
SURF5	surfeit 5 isoform a	19999	0.00024	-
DNMT1	DNA (cytosine-5-)-methyltransferase 1	15041	0.00037	-
PROM2	prominin 2	20721	0.00041	-
ORC5L	origin recognition complex subunit 5 isoform 1	18297	0.00044	-
C7orf26	hypothetical protein LOC79034	27440	0.00057	-
FGF21	fibroblast growth factor 21 precursor	16223	0.00081	-
FBXO2	F-box only protein 2	1430	0.00096	-
SOCS6	suppressor of cytokine signaling 6	1541	0.0011	-
FLJ20186	differentially expressed in FDCP 8 isoform 1	25192	0.0011	+
KCNQ1	potassium voltage-gated channel; KQT-like subfamily; member 1 isoform 1	16826	0.0011	-
TTLL3	tubulin tyrosine ligase-like family; member 3 isoform 2	3393	0.0011	-
CD209	CD209 antigen	1639	0.0011	-
GNAS	guanine nucleotide binding protein; alpha stimulating activity polypeptide 1 isoform a	7256	0.0011	+
C15orf2	hypothetical protein LOC23742	27330	0.0012	+
MYR8	myosin heavy chain Myr 8	18936	0.0012	-
COL21A1	alpha 1 type XXI collagen precursor	5188	0.0013	-
ABHD14A	abhydrolase domain containing 14A	4514	0.0014	-
GNAT1	guanine nucleotide binding protein; alpha transducing activity polypeptide 1	6788	0.0015	-
CCNA1	cyclin A1	16506	0.0015	-
SCGB1A1	secretoglobin; family 1A; member 1 (uteroglobin)	9424	0.0016	-
GPR172A	G protein-coupled receptor 172A	16693	0.0019	-
CD40	CD40 antigen isoform 1 precursor	21586	0.0020	-
CD40	CD40 antigen isoform 1 precursor	25236	0.0020	-
TMC4	transmembrane channel-like 4	25713	0.0020	-
MGMT	O-6-methylguanine-DNA methyltransferase	2946	0.0021	-
KCNB1	potassium voltage-gated channel; Shab-related subfamily; member 1	14740	0.0021	-
NR5A1	nuclear receptor subfamily 5; group A; member 1	724	0.0021	-
NMUR1	neuromedin U receptor 1	18260	0.0021	-
COG2	component of oligomeric golgi complex 2	17060	0.0022	+
C1orf22	hypothetical protein LOC80267	19913	0.0022	-
GTF3A	general transcription factor IIIA	24750	0.0022	-
ANXA9	annexin A9	20401	0.0022	-
CTSL	cathepsin L preproprotein	11068	0.0023	-
CASKIN2	cask-interacting protein 2	4171	0.0023	-
DNAI1	dynein; axonemal; intermediate polypeptide 1	8523	0.0024	-
CPNE6	copine 6	1566	0.0024	-
SCAP	SREBP cleavage-activating protein	26572	0.0024	+
TPCN2	two pore segment channel 2	22462	0.0024	-
PGAM2	phosphoglycerate mutase 2 (muscle)	26033	0.0025	-
BMPR1A	bone morphogenetic protein receptor; type IA precursor	6812	0.0025	-
APOB	apolipoprotein B precursor	5340	0.0026	-
USP4	ubiquitin specific protease; proto-oncogene isoform a	18877	0.0027	-
DKK2	dickkopf homolog 2 precursor	1409	0.0027	-
FLJ42486	hypothetical protein LOC388021	116	0.0027	+
FLJ32569	hypothetical protein LOC148811	14129	0.0027	-
FBXO17	F-box protein FBG4 isoform 2	8782	0.0027	-
CCDC28B	coiled-coil domain containing 28B	13649	0.0027	-
PDE7B	phosphodiesterase 7B	2673	0.0028	-
PSENEN	presenilin enhancer 2	7811	0.0029	-
DEFA4	defensin; alpha 4 preproprotein	19296	0.0029	-
FLJ36046	hypothetical protein LOC164592	22234	0.0030	-
ITGA8	integrin; alpha 8	13427	0.0030	-
C1orf42	chromosome 1 open reading frame 42	11694	0.0033	-
LRRC15	leucine rich repeat containing 15	26814	0.0033	-
MAGI2	membrane associated guanylate kinase; WW and PDZ domain containing 2	20484	0.0033	-
SLC7A7	solute carrier family 7 (cationic amino acid transporter; y+ system); member 7	19863	0.0034	-
WDR41	WD repeat domain 41	2049	0.0034	-
HOXA2	homeobox A2	9788	0.0035	-
BANF1	barrier to autointegration factor 1	21392	0.0037	-
LSM1	Lsm1 protein	14732	0.0037	+
WFDC12	WAP four-disulfide core domain 12 precursor	20441	0.0037	-
LITAF	LPS-induced TNF-alpha factor	8221	0.0037	-
SURF6	surfeit 6	18726	0.0037	-
PEG10	paternally expressed 10	6933	0.0038	+
FLJ30707	hypothetical protein LOC220108	3010	0.0038	-
PRLH	prolactin releasing hormone	11315	0.0039	-
MRPL18	mitochondrial ribosomal protein L18	10544	0.0039	-
C20orf141	hypothetical protein LOC128653	1274	0.0040	-
PRPF31	pre-mRNA processing factor 31 homolog	13591	0.0040	+
C6orf192	hypothetical protein LOC116843	13501	0.0040	+
ESPN	Espin	12998	0.0041	-
CX36	connexin-36	21020	0.0041	-
CGB	chorionic gonadotropin beta 3 subunit precursor	554	0.0042	-
EPB41L4B	erythrocyte membrane protein band 4.1 like 4B isoform 2	19624	0.0042	-
HLA-DRA	major histocompatibility complex; class II; DR alpha precursor	25745	0.0044	-
APOC2	apolipoprotein C-II precursor	27369	0.0044	-
MGC9850	hypothetical protein MGC9850	2325	0.0045	-
SIAHBP1	fuse-binding protein-interacting repressor isoform b	18163	0.0045	-
LOC51315	hypothetical protein LOC51315	8253	0.0047	+
FAM26C	hypothetical protein LOC255022	9095	0.0048	-
PEPD	Xaa-Pro dipeptidase	9333	0.0050	+
CEACAM4	carcinoembryonic antigen-related cell adhesion molecule 4	21516	0.0050	-

**Fig 3:**
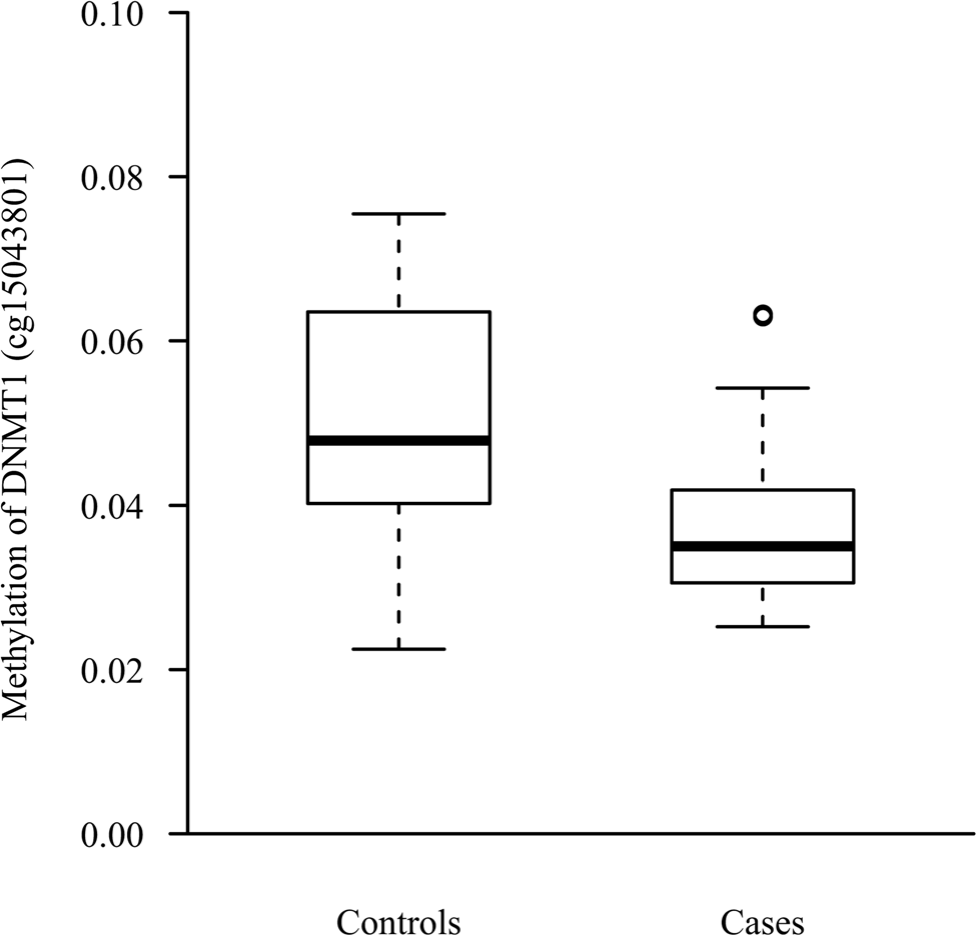
Methylation of the DNA (cytosine-5-)-methyltransferase 1 gene. Distribution of the level of methylation (β*-*value) of the *DNMT1* gene in offspring of diabetic fathers and offspring of diabetic mothers. The boxes limits represent first and third quartile of the distribution, with the median inside. Outer whiskers extend to the most extreme data point which is no more than1.5 times the interquartile range from the box. p = 0.0004 between the 2 groups.

Interrogation of the Acumenta Literature Lab did not find methylation differences in known genes and pathways involved in kidney development and function between cases and controls. Using the software with no a priori, “Methylation pathway” was identified as the only pathway strongly (p = 0.0014) associated with down-methylated sites in cases. This was due to the DNA (cytosine-5-)-methyltransferase 1 (*DNMT1*) gene which was less methylated (site cg15043801) in cases (Fig 3).

To confirm the Illumina Chip analysis, we performed DNA methylation specific PCR (Epitect, Qiagen) for 7 genes less methylated in offspring of diabetic mothers (DNMT1, TMBIM1, SOCS6, COL21A1, CCNA1, SURF5, GPR172A). We found that global methylation was lower in offspring of diabetic mothers: 0.005 (0.024–0.001) [median (75%Q—25%Q)] vs 0.013 (0.055–0.002) in offspring of diabetic fathers (p = 0.004).

We then studied possible relationships between the level of methylation in the differentially methylated sites between the 2 groups and kidney function parameters. We found an opposite correlation between the average level of methylation of the 74 sites less methylated in cases and Glomerular Filtration Rate in basal state (cases: r = 0.27 (95%CI:-0.22;0.66); controls: -0.44 (-0.75;0.03); p = 0.03 for interaction) and in response to amino acid infusion (cases: 0.10 (-0.38;0.54); controls: -0.47 (-0.77;-0.01); p = 0.06 for interaction). Although non-significant, a similar pattern of correlation was observed between the average level of methylation of the *DNMT1* gene and Glomerular Filtration Rate (basal and stimulated). No correlation was found with other kidney parameters.

## Discussion

Our results suggest that in utero exposure to hyperglycemia is associated with alterations in genome-wide DNA methylation profile. These alterations were related to kidney dysfunction in adults. We did not identify any differential methylation on genes currently known for their function in kidney development. One explanation could be that DNA methylation varies depending on cell type and we do not have evidences that DNA methylation profile in the kidney is similar to what is observed in peripheral leukocytes, except if DNA methylation changes occurred in the very early fetal development. Interestingly, one of the strongest observed associations was for the methylation of the gene encoding DNA methyltransferase 1 (*DNMT1*) which is known to be involved in early phases of development. Thus, the link between methylation alteration and kidney dysfunction may result from a nonspecific imprinting process.

Our analysis reveals mostly a lower methylation profile in cases with 74 sites less methylated and 13 sites more methylated when compared to controls. Although permutation analyses allow calculation of order statistic distributions and multiple-testing adjusted P-values [25], we cannot exclude false positive or negative results due to the small sample size and the low variation in site-specific methylation between individuals [22, 23]. Thus, we acknowledge caution in interpreting the results of differentially methylated genes and the necessity to replicate these findings in other groups of subjects.

Reduced methylation has been reported with aging [29], smoking [30], gender with unmethylated X chromosome in males [31], but also with caloric restriction during pregnancy [32] and with type 2 diabetes [33]. Our data provide for the first time evidence of site-specific fetal-hyperglycemia association for a certain number of CpG sites which are less methylated in case of fetal exposure to hyperglycemia.

The positive correlations observed in the case group between renal function and the average β-value of the 74 CpG sites down-methylated support a potential role for methylation as an epigenetic phenomenon in the programming of renal dysfunction. In rat models of dietary protein restriction, the angiotensin receptor gene 1b (*AT1b*) in the adrenal is significantly under-methylated early in offspring life, and in vitro, *AT1b* gene expression is highly dependent on promoter methylation [34]. Also, fibrogenesis in the kidney is a possible mechanism since epigenetic modifications has been shown to cause fibroblast activation [35]. Humans who were prenatally exposed to famine during the Dutch Hunger Winter in1944–45 had, 6 decades later, less DNA methylation of the imprinted *IGF2* gene compared with their unexposed, same-sex siblings [32]. Thus, renin-angiotensin system, fibrogenesis and *IGF2* gene may be targeted by epigenetic modifications participating to fetal programing of renal dysfunction. Unfortunately, in our study, none of the differentially methylated CpGs involved these genes/pathways.

Epigenetic mechanisms such as genomic imprinting may contribute to the programming of health and disease (review in [36]). While most genes are expressed from both parental loci simultaneously, some only expressed from either the maternal or the paternal allele, are called parental imprinting genes. Most imprinted genes act during fetal development, making them plausible candidate for fetal programming [37]. The gene encoding DNA methyltransferase 1 (*DNMT1*) was the methylated gene in cases that has been picked up by the automated method of literature interrogation. It is a key enzyme in maintaining methylation patterns during cell division and it plays a crucial role in maintaining methylation marks of the imprinted genes [38] and consequently their expression regulation during development. The deletion of *DNMT1* causes disruption of the maintenance imprinting leading to fetal death in rodents [39]. It has been shown that hyper-methylation of the *DNMT1* promoter is associated with its decreased expression [40]. Thus, methylation modification of *DNMT1* may represent a potential mechanism of fetal programing by hyperglycemia causing abnormal kidney development through parental imprinting. Changes in gene expression by epigenetic process are not restricted to imprinted genes. *DNMT1* is highly expressed in the kidney and Bechtel et al. demonstrated that it is involved in kidney fibrosis by hypermethylating *RASAL1*, encoding an inhibitor of the Ras oncoprotein associated with the perpetuation of fibroblast activation [35]. Lastly, it is also possible that *DNMT1* affects gene expression by mechanisms independent of DNA methylation, as it has been demonstrated earlier in lung carcinoma [41]. Studying methylation levels of some imprinted gene in new-born (cord blood) from gestational diabetes, El Hajj et al recently found an under-methylation of the maternally imprinted *MEST* gene but they did not look at *DNMT1* [42].

Whether or not fetal exposure to hyperglycemia (or its associated metabolic abnormalities) impacts tissue DNA methylation is still unanswered. To our knowledge, no data exist regarding kidney development and function. However, the relationships between hyperglycemia and DNA methylation have been studied in other tissues. Using the same Infinium Methylation assay than in our study, Volkmar et al. found 266 CpGs with lower methylation levels and only 10 hyper-methylated CpGs in islets isolated from T2D patients compared with non-diabetic individuals [43]. A subgroup of the differentially methylated genes involved pathways implicated in pancreatic β-cell survival and function. Lastly, El-Osta et al. reported that transient hyperglycemia induced histone modification on the promoter of the inflammatory gene *NFκB p65* in mice endothelial cells [44].

We cannot rule out that differences in DNA methylation between cases and controls were related to prematurity rather than fetal exposure to hyperglycemia. However, prematurity was late preterm delivery, occurred in a minority of subjects and was not associated with a low birth weight. In addition other mother environmental factors such as diet, physical exercise, body weight gain, or stress may impact DNA methylation.

In conclusion, this study is the first evidence that kidney dysfunction associated with moderate hyperglycemia (or its related metabolic alterations) during fetal development may be mediated by DNA methylation modifications. Confirmatory results in other groups of subjects are needed as well as investigating the direct biological mechanisms linking DNA methylation status and kidney function.
